# Alveolar bone loss, platelet and glycosylated haemoglobin levels in 239 patients. A clinical study

**DOI:** 10.4317/medoral.23181

**Published:** 2020-03-06

**Authors:** Mario Pérez-Sayáns, Andrés Blanco-Carrión, Abel García-García, Cintia M Chamorro-Petronacci, Karem L Ortega, Juan Suárez-Quintanilla

**Affiliations:** 1 DDS, PhD. Oral Medicine, Oral Surgery and Implantology Unit. Faculty of Medicine and Dentistry, Santiago de Compostela University; 2 Instituto de Investigación Sanitaria de Santiago (Sanitary Research Institute of Santiago) (IDIS), Santiago de Compostela, Spain; 3 MD, PhD. Oral Medicine, Oral Surgery and Implantology Unit. Faculty of Medicine and Dentistry, Santiago de Compostela University; 4 DDS, MDS, PhD. Oral and Maxillofacial Pathology, School of Dentistry, University of São Paulo, São Paulo, Brazil; 5 PhD, MD. Anatomy Department, Santiago de Compostela University. Primary care dentist SERGAS (Galician Health Service)

## Abstract

**Background:**

The relation between periodontal disease and systemic pathologies is still not widespread among general practitioners. The aim of our study is to evaluate whether or not periodontal radiological diagnosis can aid the detection of blood alterations associated with acquired systemic diseases.

**Material and Methods:**

This is a cross sectional study. All of the participants underwent a panoramic radiograph and a complete blood test. Morphological bone loss was considered as positive in those patients who showed radiographically more than 1 tooth with bone loss greater than or equal to the middle third of the root. The statistical analysis was performed by comparing the variables using the ANOVA or U-Mann-Whitney tests for independent samples with normal conditions. The correlation coefficient was analysed using the Pearson test.

**Results:**

239 patients were included in our study (96 men and 143 women) with an average age of 64.40 years. 59.04% of the patients were determined as morphological bone loss positive and had on average 4 teeth less than negative patients (*p* <0.0001). Also the average platelet levels in positive patients were lower (*p* = 0.024) and mean levels of HBA1c (*p* = 0.009) were higher.

**Conclusions:**

Morphological bone loss parameter can be useful both for dentists and general practitioners to refer, subsequently, to periodontal specialist.

** Key words:**Periodontal diseases, blood chemical analysis, blood platelets, alkaline phosphatase, glycosylated haemoglobin A, alveolar bone loss.

## Introduction

Periodontal disease (PD) is a chronic inflammatory process that damages the tissues surrounding the teeth, causing the progressive loss of the alveolar bone, which, in very advanced or very aggressive cases induces the loss of teeth, and, from an anatomical point of view causes the destruction of the ligament of the alveolodental gomphosis (1). PD, and dental caries are the most commonly diagnosed and treated pathologies in dental consultations, but from a medical point of view, PD is considered to be a chronic inflammatory disease (2). That is to say that it is categorised as a disease in which the body's defence system alters the cellular DNA mechanisms due to excessive inflammation time, given that these are designed to repair tissues with short-term anti-inflammatory mechanisms.

For this reason, the manner in which chronic PD is evaluated differs in dental and medical practice. In dental practice the disease must be measured in order to assess its evolution in each tooth by implementing techniques which require a complex instruction such as the periodontal pocket probing depth, the clinical insertion loss, bleeding in probing or the loss of alveolar bone by means of a radiological study. However, there is no consensus as to which is the best method to use. These parameters are used, not only to diagnose the disease but also to assess both its progression following the treatment phases and the need for more complex and invasive treatments (3).

However, in medical practice it is easy to evaluate the presence of chronic inflammatory periodontal disease by means of a panoramic radiography (OPG). This procedure involves measuring the alveolar bone loss in the tooth which has the greatest loss out of all of those present. The instruction given to doctors for this method is simple and very useful as all patients who present with more than 1/3 of alveolar loss are classified as having chronic inflammatory disease (4).

There is considerable evidence regarding the association between PD and systemic diseases such as renal dysfunction, diabetes mellitus, rheumatoid arthritis, Alzheimer's disease, cardiovascular risk, obesity, or cerebrovascular disease (5,6). The relationship between PD and these systemic diseases is bidirectional meaning therefore that PD can cause adverse results and that certain systemic diseases can predispose a person to have periodontitis (3). This relationship is explained by the elevation of pro-inflammatory markers in the bloodstream that are observed in periodontal infection, which will decrease following periodontal therapy, and which can result, for example, in insulin resistance (7). Likewise, the bacterial colonisation of PD-causing bacteria such as P.gingivalis, *T. forsythia* and *A. actinomycetecomitans*, were found in atheromatous plaques (3). Another association which has undergone considerable study is the relationship between PD and the delivery of a preterm infant (8).

However, despite the prevalence of PD, there is still not enough awareness amongst the majority of general practitioners worldwide who are not specialised in the oral cavity with regards to its relationship with systemic diseases and the need to treat affected patients (9,10). It is therefore necessary for doctors and other health professionals to educate patients regarding this relationship and to recommend them dental care and oral health restoration procedures given that the evidence suggests that the treatment of PD leads to an improvement in the systemic pathology (3).

The aim of our study is to evaluate whether or not the radiological diagnosis of PD can aid the detection of blood alterations associated with acquired systemic diseases.

## Material and Methods

- Study design

It is a cross sectional study. The calculation of the sample size was estimated in relation to the patients treated in the Oral Health Service (the target population covers the probability of the disease). Thus, for a general population of health coverage of 34000 inhabitants, with an annual health care of 2600 patients, with about 300 first consultations (study inclusion criteria), for a two-year study, assuming heterogeneity of 50%, margin error of 5% and confidence level of 95%, the appropriate sample size would be 235 patients. This sample size has been calculated with Epidat 2.4 (SERGAS, Galicia, Spain).

The exposition of this study has been designed according to the STROBE recommendations for observational studies. The data was collected from January 2017 to January 2019. All of the procedures were carried out with the proper understanding and written consent of the subjects in accordance with the Declaration of Helsinki and its subsequent modifications.

- Study inclusion/exclusion criteria

Inclusion criteria: All patients, over the age of 18 who attended a primary dental care service in Santiago de Compostela, Spain and who had not previously been diagnosed with PD were included systematically. All of the patients who decided to participate in the study signed an informed consent form and they underwent a OPG and a complete blood analysis.

Exclusion criteria: Patients under the age of 18, pregnant patients, those who had previously been treated periodontally, and those who refused to sign the informed consent form were excluded.

- Radiographic study

OPGs were performed using an orthopantomographer with radiographic values of 65Kv and 6mA. The images were saved and managed with the Sidexis® Software (Dentsply Sirona Inc., Pennsylvania, USA). The OPG allowed for the evaluation of the number of teeth present as well as the overall alveolar bone loss. An anatomical morphological criterion was followed in which it is considered that there is positive morphological bone loss (MBL) when there is more than 1 tooth with loss greater than or equal to the middle third of the radicle, as previously recommended by other authors (4).

- Evaluation of cases

Two specialised dentists (MPS and CCP) and a primary care physician (JSQ) were trained to analyse the radiographs. Two evaluators evaluated, in a pilot study of 20 radiographs, the existence of morphological bone loss, the agreement between the two independent evaluators, was 100%.

- Data and variables

The patients' data was collected from their clinical records in a database specifically designed for that purpose. The data was analysed anonymously by assigning a code to each patient so that the data could not be associated with a specific patient. Two independent researchers input the patient data, in duplicate, in order to eliminate any potential errors. The clinical and radiological variables collected for each patient were: age, gender, systemic diseases, presence or absence of MBL, and number of missing teeth (without taking into account third molars).

- Blood study

With regards to the blood study, the levels of the following elements were considered: Leukocytes, Red Blood Cells, Haemoglobin, Haematocrit, Platelets, Neutrophils, Lymphocytes, Monocytes, Eosinophils, Basophils, ESR (Erythrocyte Sedimentation Rate after one hour), Prothrombin Time, INR, Fibrinogen, Glucose, Urea, Uric Acid, Creatinine, Sodium, Potassium, Bilirubin, AST / GOT, ALT / GPT, GGT, Alkaline Phosphatase, Total Proteins, Albumin, Calcium, Cholesterol, Triglycerides, HDL, LDL, Thyrotropin (TSH), Glycosylated Haemoglobin A1c (HBA1c).

- Statistical analysis

Descriptive statistics were performed using frequencies and percentages for categorical variables, and means and standard deviations were taken for quantitative variables. The normality of the variables was verified by the Kolmogorov-Smirnov test. The contingency Tables were constructed using the chi-square test. The statistical analysis was performed by comparing the variables using the ANOVA or U-Mann-Whitney tests for independent samples with normal conditions. The correlation coefficient was analysed using the Pearson test. Correlation analysis were performed to verify that the analytical and analytical results make sense biomedical and clinical. All of the differences in which the value of *p* was less than or equal to 0.05 were considered to be statistically significant.

## Results

A total of 239 patients were included in this study. The final sample consisted of 96 men and 143 women with an average age of 64.40 years (SD = 12.66) and an age range of between 40 and 91 years old. We observed that 59.04% of the patients had MBL. The descriptive data and the analysis of the relationship of the variables of the study with bone loss can be seen in Table 1.

**Table 1:**
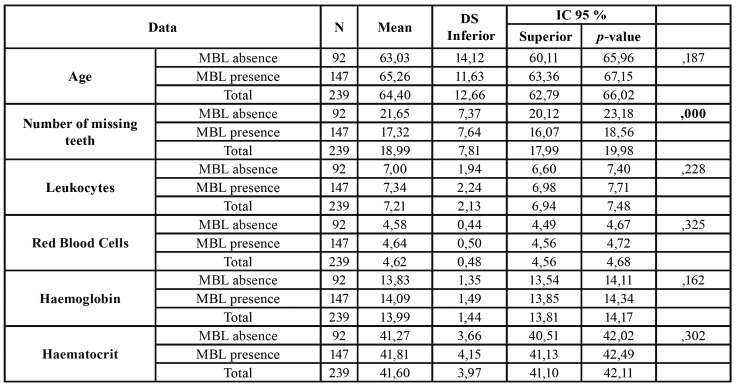
Descriptive data and relationship analysis of study’s variables with bone loss.

**Table 1 cont.**
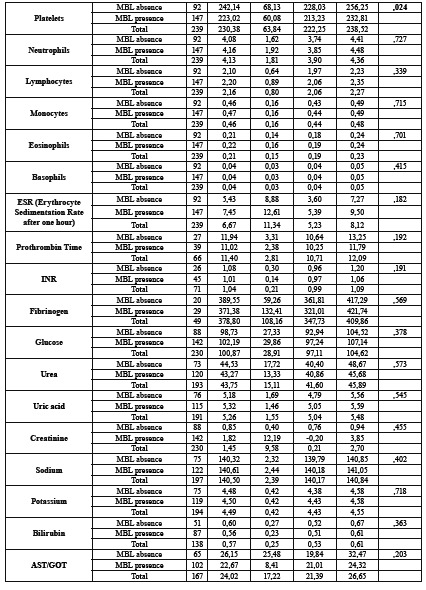
Descriptive data and relationship analysis of study’s variables with bone loss.

**Table 1 cont.**
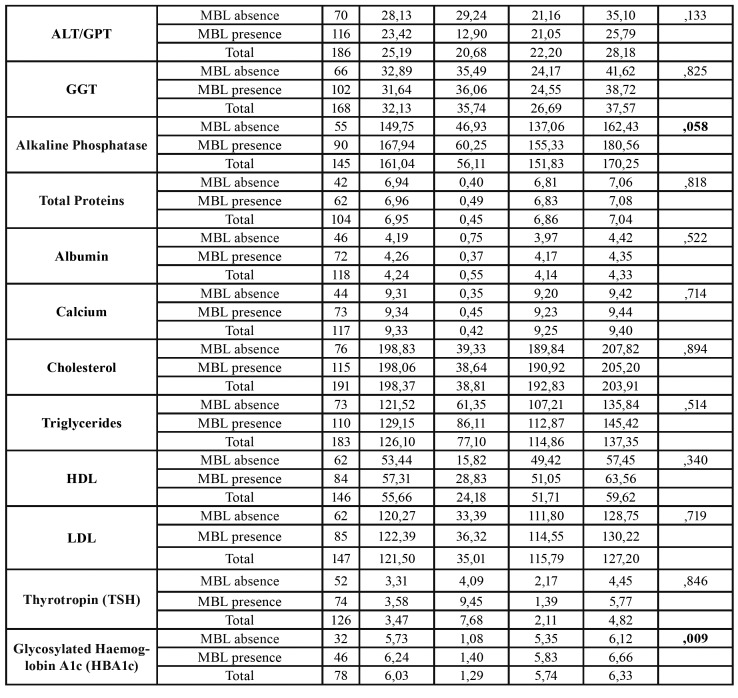
Descriptive data and relationship analysis of study’s variables with bone loss.

Patients with positive MBL had an average of 4 teeth less (17.32 teeth (SD = 7.64)) than patients with negative MBL (21.65 teeth (SD = 7.37)) (*p* <0.0001). In a bivariate analysis we found statistically significant results which demonstrated that patients who presented a visible MBL on the OPG had lower mean platelet levels 223.02 u / microl (SD = 60.08) compared to 242.14 u / microl (SD = 68.13) (*p* = 0.024). Likewise, patients suffering from MBL had higher mean levels of HBA1c, 6.24 IU / ml (SD = 1.40) compared to 5.73 (SD = 1.08) (*p* = 0.009). The mean age of patients with MBL was 2 years greater, 65.26 years (SD = 11.63) compared to 63.03 years (SD = 14.12) but there were no statistically significant differences. We found that the alkaline phosphatase levels were higher in patients with MBL, 167.94 IU / l (SD = 60.25) compared to 149.75 IU / l (SD = 46.93) but there were no statistically significant differences (*p* = 0.058). We did not find any other relationship between MBL and blood parameters or clinical variables.

The correlation study reveals that there are numerous statistically significant correlations (Table 2), although we can highlight those in which the correlation coefficient (CC) is above 0.4 (moderate positive correlation). Therefore, age and teeth correlated inversely (CC = -0.434; *p* <0.001), age and urea correlated directly (CC = 0.464; *p*<0.001), HbA1c and glucose correlated positively (CC = 0.845, *p*<0. 001) and HbA1c and sodium correlated inversely (CC = -0.492; *p*<0.001).

**Table 2:**
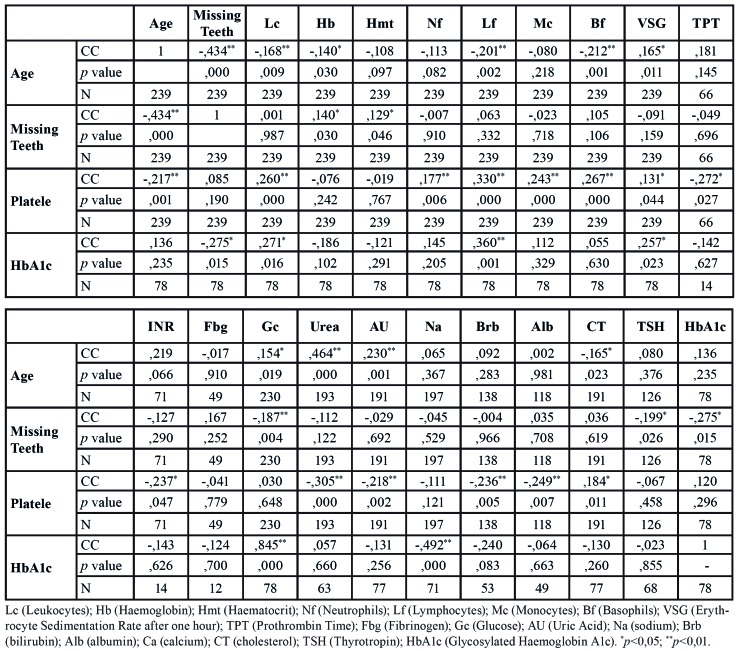
Correlation study of the mean variables.

## Discussion

A total of 239 patients were included in this study, and more than half, 59.04%, presented with MBL. This prevalence is considerably greater than the results recorded in other studies within same geographic territory where a prevalence of 30% is estimated (11).

The different morphometric indices which are measurable in OPG have been studied for the identification of PD (12,13) and other diseases such as osteoporosis, sickle cell disease or macular degeneration (14,15). This can be explained by the fact that many systemic pathologies share risk factors (as smoking, diet, etc.) with PD, and may have similar etiologic mechanisms.

The statistical analyses of our study reveal an association between the MBL and the number of teeth, as well as the age and the number of teeth. Taking into account that the final consequence of PD is the loss of teeth, it seems evident that over time the number of teeth of patients diagnosed with periodontitis will decrease.

Another significant finding from our analysis was the association of lower levels of platelets in patients with MBL. The main function of platelets is haemostasis, but these also promote chronic inflammatory reactions, such as atherosclerosis, modulate acute inflammatory processes (16) such as infections, and contribute to the exacerbation of autoimmune conditions such as asthma or arthritis (17). There is a clear relationship between changes in the platelet count and its function with age. Although the platelet response increases with age, the count decreases in patients aged over 60 (18). This coincides with our findings given that the mean age of our patients was 64.40 years, from an age range of 40-91 years, and statistical analyses showed an inverse correlation between platelet count and age.

A positive correlation of platelets and leukocytes has also been observed in this study. Maybe, this result is determined by the fact that platelets, through the expression of toll-like receptors, mediate the inflammatory response by interacting with leukocytes. Although a cause effect relationship cannot be warranted, previous studies have also observed this association in patients with PD (19).

Caula *et al*. discovered an association between increased alkaline phosphatase and blood creatinine and PD (20). We did not find differences in creatinine concentration between patients with positive MBL and negative MBL. We did find differences in the concentration of alkaline phosphatase, although this was not statistically significant (*p* = 0.058). Perhaps this is due to the fact that through the MBL criterion we have not assessed the severity of PD, a condition that Caula *et al*. took into account when studying the relationship. De *et al*. analysed alkaline phosphatase in patients with chronic PD, with and without type 2 diabetes mellitus, finding a significantly higher concentration in the first group (21). Jeyasree *et al*. also found a decrease in alkaline phosphatase in blood following the first phase of periodontal treatment in patients with chronic periodontitis (22).

Positive MBL patients have presented significantly higher average HbA1C levels. The prevalence of patients with PD is significantly higher in individuals with diabetes or with a precondition to suffer from diabetes (23). The coagulation cascade dysfunction has been widely studied in diabetic patients. In type 2 diabetes platelets adhere to the vascular endothelium and aggregate more rapidly than in healthy individuals (24,25). Coinciding with our previous results, platelets would initiate a vascular adhesion process and the amount of circulating platelets would decrease. Many randomised studies have attempted to associate the deregulation of HbA1C, either as a diagnostic marker between healthy patients and patients with PD, or with the same patients before and after periodontal treatment (26). Numerous studies associate the presence of PD with an increased risk of type 2 diabetes, and longitudinal studies reveal a reduction in HbA1C of 0.27-0.48% following periodontal therapy after 3 months of follow-up (26). However, although all studies show some effect, not all of the results are statistically significant (3,27,28), and we cannot forget that many systemic diseases share etiological mechanisms that have not been studied in depth in these analysis.

Our findings coincide with the study by Montero *et al*. (29) who developed a predictive model for severe and moderate periodontitis in Americans between 2011 and 2012. More than 3000 patients were included in this study, and age, gender, ethnicity, smoking habits and HbA1C were identified as variables for the predictive model of the disease, reaching a sensitivity of 70% and a specificity of 67.6 %.

Given its asymptomatic character, except in advanced stages, the identification of PD is sometimes conflicting, but taking into account the prevalence of the disease it seems clear that there is an infra diagnosis of this disorder. The use of the MBL is simple, it does not require additional instruments (specific periodontal instruments, periodontal tests for bleeding and bacterial plaque, etc.), and it is easily applied by the health professional. The MBL can be an additional tool when the OPG is taken for medical diagnosis and to evaluate paradental bone margin. The objective of using OPG by general practitioners, does not seek the diagnosis and accurate classification of the PD suffered by the patient, but a general screening to be able to subsequently refer to the dentist or periodontal specialist. Parameters such as retraction, depth of probing, loss of insertion and periodontal probing technique are essential for the specific diagnosis of PD, and require deeper and more complex knowledge than the generic use of OPG.

Due to the prevenTable and treaTable nature of periodontitis, subjects diagnosed with periodontitis should be informed and treated so that the bacterial load and the hyperproduction of pro-inflammatory cytokines can be reduced, thus promoting a better quality of life, especially in patients with advanced age (30). Cases of dementia have expanded among the elderly population. The number of patients with dementia is expected to triple by 2050, and most cases of dementia are associated with Alzheimer's disease. Through this approach there is a special interest in identifying modulate risk factors (such as PD) for dementia and Alzheimer's disease since it is estimated that these factors can contribute to 30-50% of cases (30).

Limitations in our study include the lack of classification between the types of PD, and the lack of analysis on the active state of PD, which, as we have seen, could influence the alteration of markers. Other limitation of our study is that OPG is not useful to detect PD activity, since it shows more historical aspects associated with destruction. Radiographic alterations produced by PD implies a late diagnosis. In this study bone loss associated with endodontic pathology, possible dental fissure or occlusal trauma has not been taken into account. It is important to remark that this is a cross-sectional study and relationships between variables cannot assure a cause-effect relation.

In view of the foregoing, the incorporation of the MBL parameter into our dental care and primary medical care protocol may be useful both for dentists and primary care physicians.

The statistical analysis revealed association between the MBL and variables as lower levels of platelets, higher average HbA1C levels, the number of teeth, and also the age and the number of teeth. This work provides data that allows establishing a line of work that attempts to correlate diagnosed PD with blood disorders due to systemic diseases.
